# Immunoproteasome deficiency results in age-dependent development of epilepsy

**DOI:** 10.1093/braincomms/fcae017

**Published:** 2024-01-29

**Authors:** Hanna Leister, Felix F Krause, Beatriz Gil, Ruslan Prus, Inna Prus, Anne Hellhund-Zingel, Meghma Mitra, Rogerio Da Rosa Gerbatin, Norman Delanty, Alan Beausang, Francesca M Brett, Michael A Farrell, Jane Cryan, Donncha F O’Brien, David C Henshall, Frederik Helmprobst, Axel Pagenstecher, Ulrich Steinhoff, Alexander Visekruna, Tobias Engel

**Affiliations:** Institute for Medical Microbiology and Hygiene, Philipps-University, 35043 Marburg, Germany; Institute for Medical Microbiology and Hygiene, Philipps-University, 35043 Marburg, Germany; Department of Physiology and Medical Physics, Royal College of Surgeons in Ireland, University of Medicine and Health Sciences, D02 YN77 Dublin, Ireland; Department of Physiology and Medical Physics, Royal College of Surgeons in Ireland, University of Medicine and Health Sciences, D02 YN77 Dublin, Ireland; Department of Physiology and Medical Physics, Royal College of Surgeons in Ireland, University of Medicine and Health Sciences, D02 YN77 Dublin, Ireland; Institute for Medical Microbiology and Hygiene, Philipps-University, 35043 Marburg, Germany; Department of Physiology and Medical Physics, Royal College of Surgeons in Ireland, University of Medicine and Health Sciences, D02 YN77 Dublin, Ireland; FutureNeuro, SFI Research Centre for Chronic and Rare Neurological Diseases, Royal College of Surgeons in Ireland, University of Medicine and Health Sciences, D02 YN77 Dublin, Ireland; FutureNeuro, SFI Research Centre for Chronic and Rare Neurological Diseases, Royal College of Surgeons in Ireland, University of Medicine and Health Sciences, D02 YN77 Dublin, Ireland; Department of Neurology, Beaumont Hospital, D09V2N0 Dublin, Ireland; Department of Neuropathology, Beaumont Hospital, D09V2N0 Dublin, Ireland; Department of Neuropathology, Beaumont Hospital, D09V2N0 Dublin, Ireland; Department of Neuropathology, Beaumont Hospital, D09V2N0 Dublin, Ireland; Department of Neuropathology, Beaumont Hospital, D09V2N0 Dublin, Ireland; Department of Neurosurgery, Beaumont Hospital, D09V2N0 Dublin, Ireland; Department of Physiology and Medical Physics, Royal College of Surgeons in Ireland, University of Medicine and Health Sciences, D02 YN77 Dublin, Ireland; FutureNeuro, SFI Research Centre for Chronic and Rare Neurological Diseases, Royal College of Surgeons in Ireland, University of Medicine and Health Sciences, D02 YN77 Dublin, Ireland; Institute of Neuropathology, Philipps-University, 35043 Marburg, Germany; Core Facility for Mouse Pathology and Electron Microscopy, Philipps-University, 35043 Marburg, Germany; Institute of Neuropathology, Philipps-University, 35043 Marburg, Germany; Core Facility for Mouse Pathology and Electron Microscopy, Philipps-University, 35043 Marburg, Germany; Institute for Medical Microbiology and Hygiene, Philipps-University, 35043 Marburg, Germany; Institute for Medical Microbiology and Hygiene, Philipps-University, 35043 Marburg, Germany; Department of Physiology and Medical Physics, Royal College of Surgeons in Ireland, University of Medicine and Health Sciences, D02 YN77 Dublin, Ireland; FutureNeuro, SFI Research Centre for Chronic and Rare Neurological Diseases, Royal College of Surgeons in Ireland, University of Medicine and Health Sciences, D02 YN77 Dublin, Ireland

**Keywords:** immunoproteasome, brain, ageing, epilepsy

## Abstract

The immunoproteasome is a central protease complex required for optimal antigen presentation. Immunoproteasome activity is also associated with facilitating the degradation of misfolded and oxidized proteins, which prevents cellular stress. While extensively studied during diseases with increasing evidence suggesting a role for the immunoproteasome during pathological conditions including neurodegenerative diseases, this enzyme complex is believed to be mainly not expressed in the healthy brain. In this study, we show an age-dependent increase in polyubiquitination in the brains of wild-type mice, accompanied by an induction of immunoproteasomes, which was most prominent in neurons and microglia. In contrast, mice completely lacking immunoproteasomes (triple-knockout mice), displayed a strong increase in polyubiquitinated proteins already in the young brain and developed spontaneous epileptic seizures, beginning at the age of 6 months. Injections of kainic acid led to high epilepsy-related mortality of aged triple-knockout mice, confirming increased pathological hyperexcitability states. Notably, the expression of the immunoproteasome was reduced in the brains of patients suffering from epilepsy. In addition, the aged triple-knockout mice showed increased anxiety, tau hyperphosphorylation and degeneration of Purkinje cell population with the resulting ataxic symptoms and locomotion alterations. Collectively, our study suggests a critical role for the immunoproteasome in the maintenance of a healthy brain during ageing.

## Teaser

Leister *et al*. report that immunoproteasome deficiency results in spontaneous seizures and other brain pathologies such as tau hyperphosphorylation, degeneration of Purkinje cells and increased anxiety in aged mice. This suggests that the upregulation of immunoproteasomes during ageing affords protection from the exaggerated accumulation of protein aggregates and epilepsy development.

## Graphical Abstract

**Graphical Abstract:**
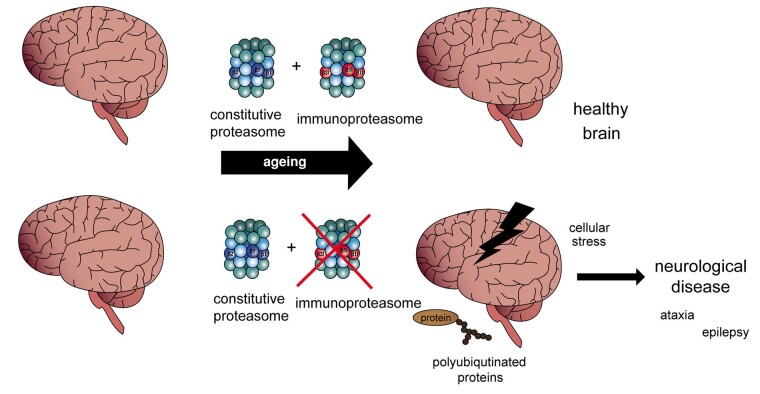

## Introduction

The proteasome is an evolutionary conserved protease that recognizes and degrades damaged and misfolded proteins via proteolysis.^1^ The 26S proteasome consists of the 19S regulatory subunit and the core proteolytic enzyme, 20S proteasome.^2^ The proteasome-dependent degradation of tagged, polyubiquitinated proteins is an essential cellular pathway that regulates a wide range of cellular processes, including cell cycle, apoptosis, oxidative stress, cell proliferation and activation of transcription factors such as NF-κB.^3^ In contrast to the ubiquitously expressed constitutive proteasome, which is the crucial cellular protease containing the three catalytic subunits, β1, β2 and β5, the immunoproteasome is induced via *de novo* assembly upon stimulation of cells by Type I and Type II interferons. The incorporation of newly synthetized catalytic subunits, β1i/LMP2, β2i/MECL-1 and β5i/LMP7, is an essential cellular strategy to eliminate intracellular bacteria and viruses.^4^ The immunoproteasome exhibits an altered proteolytic function that is required for optimal generation of epitopes for presentation on major histocompatibility complex Class I (MHC-I) molecules in infected cells. It was shown that immunoproteasomes transiently replace constitutive proteasomes during an anti-bacterial (*Listeria monocytogenes*) and anti-viral (lymphocytic choriomeningitis virus) immune response in the liver.^5^ Apart from their function in the generation of a broad pool of MHC-I ligands and in triggering effective activation of cytotoxic T lymphocytes, immunoproteasomes appear to act as a pro-inflammatory factor that is involved in tissue inflammation and damage, as well as in regulation of CD4^+^ T-cell differentiation during chronic inflammatory and autoimmune disorders, such as rheumatoid arthritis and inflammatory bowel disease.^6-8^ Moreover, the potential role of immunoproteasomes in efficient degradation and removal of oxidatively damaged proteins in non-immune cells has been postulated.^9^ Emerging evidence also suggests beneficial effects of the immunoproteasome in brain diseases, such as neurodegenerative diseases (e.g. Parkinson’s, Alzheimer’s and Huntington’s diseases)^10^ and epilepsy.^11^ Whether immunoproteasome activity is essential for normal physiological processes in the brain and impacts on normal brain function remains to be determined.

In this study, we describe an induced activity of immunoproteasomes in neurons of otherwise healthy aged brains, which is evolved independently of their classical role in antigen presentation. While the brain cells of young mice express primarily constitutive proteasomes, we observed the induction of immunoproteasomes in aged brains. The deletion of all three immunoproteasome subunits led to the development of age-dependent spontaneous seizures and to a more severe kainic acid (KA)-induced status epilepticus (SE). Immunoproteasome expression was reduced in the brains of patients with epilepsy, further suggesting a role during brain hyperexcitability. Besides the generation of seizures, aged mice deficient in the immunoproteasome also showed increased tau hyperphosphorylation, neurodegeneration and anxiety, as well as motor deficits, demonstrating the essential role of the immunoproteasome in the prevention of brain damage with the resulting neurological disorders.

## Materials and methods

### Human brain tissue

This study was approved by the Ethics (Medical Research) Committee at Beaumont Hospital, Dublin (05/18), and written informed consent was obtained from all patients. The patients (*n* = 10; 5 males/5 females, Supplementary Table 1) were referred for a surgical resection of the temporal lobe for the treatment of intractable temporal lobe epilepsy (TLE). After a right temporal lobectomy, cortex samples (*n* = 10; 5 males/5 females, Supplementary Table 1) were frozen in liquid nitrogen and stored at −80°C until use. The control (autopsy) temporal cortex (*n* = 19; 10 males/9 females; Supplementary Table 1) was obtained from individuals from the Brain and Tissue Bank for Developmental Disorders at the University of Maryland, Baltimore, MD, USA.

### Experimental animals

Mice were bred and maintained under specific pathogen-free conditions at the Biomedical Research Center, Philipps-University of Marburg. Wild-type (WT), LMP7^−/−^ and LMP2^−/−^MECL-1^−/−^LMP7^−/−^ triple-knockout (TKO) mice on C57BL/6 background were used for animal experiments. The WT mice were obtained from The Jackson Laboratory. The TKO mice were kindly provided by Kenneth Rock (University of Massachusetts Medical School) and Regeneron Pharmaceuticals. All animal studies adhered to the principles of the European Communities Council Directive (2010/63/EU). All relevant national licences were reviewed and approved by the RP Gießen, Germany (Project no.: G24-2019), and by the Research Ethics Committee of the Royal College of Surgeons in Ireland (REC 1322) and the Irish Products Regulatory Authority (AE19127/P038).

### Seizure susceptibility test

SE was induced by an i.p. injection of 10 mg/kg KA (Sigma-Aldrich, Cat. no. 58002-62-3). EEG was recorded from three cranial implanted electrodes, two overlaying each dorsal hippocampus and one above the frontal cortex as reference, for 60 min. Behavioural seizures were scored using a modified Racine scale^12^: Score 1, immobility and freezing; Score 2, forelimb and/or tail extension, rigid posture; Score 3, repetitive movements, head bobbing; Score 4, rearing and falling; Score 5, continuous rearing and falling; Score 6, severe tonic–clonic seizures. Mice were scored in 5 min. intervals for 60 min after KA injection. The highest score attained during the 5 min period was recorded. The EEG signal, recorded from the skull-mounted electrodes, was analysed using an Xltek recording system (Optima Medical Ltd, Guildford, UK).

### Open field analysis

For the open field study, mice were placed in a 40 × 40 × 30 cm box and recorded for 10 min. Movements during trails were tracked and analysed by using the ANY-maze video tracking system (Version 6.32). Briefly, the box was parted into a corner zone and a centre zone to calculate how much time the mice spend in the different regions. Additionally, the speed and the entries into the different regions were captured.

### Gait analysis

To quantify gait abnormalities, the feet of the mice were painted with a non-toxic washable paint using two contrasting colours (red for forelimbs, blue for hindlimbs). Then, the mice were allowed to walk through a tunnel (7 cm wide, 8 cm high, 33 cm long) on a sheet of paper (10 × 40 cm, smooth and thick watercolour paper). The stride wide, stride length and toe spread of each mouse was measured to detect changes in gait. Only steps that were consistently spaced with clear, non-smudged footprints were used for scoring. A total of 4–6 steps per foot were analysed. The analysis was performed following previously published protocol.^13^

### Quantitative real-time polymerase chain reaction

Organs were homogenized using TRIzol Reagent. After homogenization, samples were centrifuged at 12 000*g* for 10 min and the supernatant containing RNA was transferred to an RNase-free tube. Phase separation was performed by adding chloroform (200 µl/1 ml TRIzol). Following the centrifugation at 12 000*g* for 5 min, the upper phase containing the RNA was transferred into a new tube and overlaid with isopropanol to precipitate the RNA. After centrifugation at 12 000*g* for 10 min, the supernatant was discarded and the pellet was overlaid with 75% ethanol. After another centrifugation step, the pellet was dried and resuspended in RNA-free water. Concentrations were determined using a NanoDrop spectrophotometer. cDNA synthesis was performed using the RevertAid First Strand cDNASynthesis Kit (Thermo Fisher Scientific), according to the manufacturer’s instructions. A quantitative real-time polymerase chain reaction was conducted using a 500 ng template on a StepOne Plus device (Applied Biosystems). For the analysis, the Takyon ROX SYBR Master Mix blue dTTP Kit (Eurogentec) was used. For mRNA quantification, mRNA expression was normalized to the housekeeping gene HPRT using the 2^−ΔΔ*Ct*^ method. The murine primers used are as follows: *Hprt1* fwd 5′-CTG GTG AAA AGG ACC TCT CG-3′, rv 5′-TGA AGT ACT CAT TAT AGT CAA GGG CA-3′, *Psmb8* fwd 5′-TGC TCG AGA TGT GAT GAA GG-3′, rv 5′-TGT AAT CCA GCA GGT CAG CA-3′, *Ddit3* fwd 5′-AAC TGG GGG TTT GTA TGC CTC-3′, rv 5′-ACG CAG GGT CAA GAG TAG TG-3′, *Psmb9* fwd 5′-CAT CAT GGC AGT GGA GTT TGA-3′, *Psmb9* rev 5′-ACC TGA GAG GGC ACA GAA GAT-3′.

### Western blotting

Whole cell lysates from murine and human hippocampus were generated by adding a RIPA lysis buffer (Sigma-Aldrich) supplemented with protease inhibitors (Protease Inhibitor Cocktail, Thermo Fisher Scientific). The amount of total protein was quantified with a Pierce BCA Protein Assay (Thermo Fisher Scientific). Twenty micrograms of total protein per sample were loaded on 12% sodium dodecyl sulfate–polyacrylamide gel electrophoresis gels and separated by electrophoresis. Afterwards, the proteins were transferred onto a polyvinylidene difluoride (PVDF) membrane (Bio-Rad Laboratories) and blocked with 5% bovine serum albumin (BSA) for 1 h at room temperature. The primary antibody was incubated overnight at 4°C. The primary antibodies used are as follows: anti-PSMB8/LMP7 (D1K7X, Cell Signaling, Cat. no. 13635), anti-PSMB9 (Proteintech, Cat. no. 14544-1-AP), anti-ubiquitin (eBioP4D1, eBioscience, Cat. no. 14-6078-82), anti-PSMA3 (Cell Signaling, Cat. no. 2456) and anti-PSMB5 (Proteintech, Cat. no. 19178-1-AP). Afterwards, the samples were incubated with secondary antibodies for 2 h at room temperature (RT). HRP-linked anti-rabbit IgG (Cell Signaling, Cat. no. 7074) and anti-mouse IgG (Cell Signaling, Cat. no. 7076) were used. Monoclonal anti-β-actin (Sigma-Aldrich, Cat. no. A5441) was used as the loading control. The proteins were detected using Western Blotting Luminol Reagent (Santa Cruz Biotechnology, Cat. no. sc-2048) on the MicroChemi high-performance imager (DNR Bio-Imaging Systems). Quantification was assessed using ImageJ software. The samples were normalized to the amount of β-actin.

### Immunoflourescence staining

Mice were transcardially perfused with phosphate-buffered saline (PBS) (10 ml) and 4% paraformaldehyde (PFA) (10 ml). Their brains were removed and fixed for 24 h in 4% PFA at 4°C. Afterwards, the brains were transferred to PBS and immersed in 4% agarose. Sagittal sections (30 µm) were cut using a VT1000S vibratome (Leica Biosystems, Wetzlar, Germany) and sections were stored in glycol at −20°C. The sections were placed in PBS to remove the cryosolution and incubated in 0.1% Triton in PBS for 15 min. Afterwards, the brains were transferred into glycine (1 M) for 30 min and rinsed with PBS for 10 min. After 45 min of blocking in 1% BSA in PBS, the primary antibody was incubated overnight in a blocking solution at 4°C. Subsequently, slices were rinsed with the blocking solution for 5 min and incubated with the primary antibody for 2 h at RT. After washing twice with PBS for 5 min, the slices were incubated with the secondary antibodies for 2 h at RT in the dark. Then, the samples were washed twice with PBS and water. Finally, the slices were embedded with a mounting medium containing 4′,6-diamidino-2-phenylindole (DAPI) and sealed with a coverslip. The primary antibodies used are as follows: anti-PSMB8 (D1K7X, Cell Signaling, Cat. no. 13635), anti-NeuN (A60, Merck Millipore, Cat. no. MAB377) and anti-phospho-tau (AT8, Ser 202/Thr205, Invitrogen, CA, USA, Cat. no. MN1020).

### Immunohistochemistry

Fixation and preparation of tissues were performed according to the published protocol.^14^ For immunohistochemistry (IHC), tissue samples were cut into 3 µm thick sections from formalin-fixed paraffin-embedded tissues. IHC staining was performed using a Bond Max automated staining system (Leica) with the antibodies anti-PSMB8 (D1K7X, Cell Signaling, Cat. no. 13635) and anti-Calbidin D28k (CB300, Swant Inc., Cat. no. 300). Images were acquired using the Leica Aperio Versa slide scanner and Leica Aperio eSlide Manager software v. 1.0.3.37. IHC images were analysed quantitatively using the Aperio ImageScope software v. 12.3.2.8013.

### Diaminobenzidine staining

Mice were perfused with 4% PFA and their brains were extracted and post-fixed for 24 h. The brains were then transferred to PBS and immersed in 4% agarose before being cut on a microtome into 30 µm thick sections. Next, brain sections were pre-treated for 1 h with 1% bovine serum albumin, 5% foetal bovine serum and 0.2% Triton™ X-100 and then incubated with the primary phospho-tau antibody AT8 (Invitrogen). Finally, the brain sections were incubated in the avidin–biotin complex using the Elite® VECTASTAIN® kit (Vector Laboratories). Chromogen reactions were performed with diaminobenzidine (Sigma-Aldrich) and 0.003% hydrogen peroxide for ∼10 min. The sections were coverslipped with Fluorosave™.

### Statistical analysis

For all experiments with two groups, mean values were compared using an unpaired *t*-test (GraphPad Prism 9.1.0). Multiple groups were analysed using one-way ANOVA (GraphPad Prism 9.1.0). For the survival examination of mice, survival curves were analysed by performing a Mantel–Cox test (GraphPad Prism 9.1.0). *P*-values of <0.05 were considered significant. The following *P*-values were used: *P = 0.01–0.05; **P = 0.001–0.01; ***P < 0.001. Data are presented as mean ± SD. The *n* values, representing the exact number of mice used for the experiments, are indicated in the figure legends.

## Results

### Immunoproteasomes regulate polyubiquitination and protein homeostasis in the brains of aged mice

**Figure 1:**
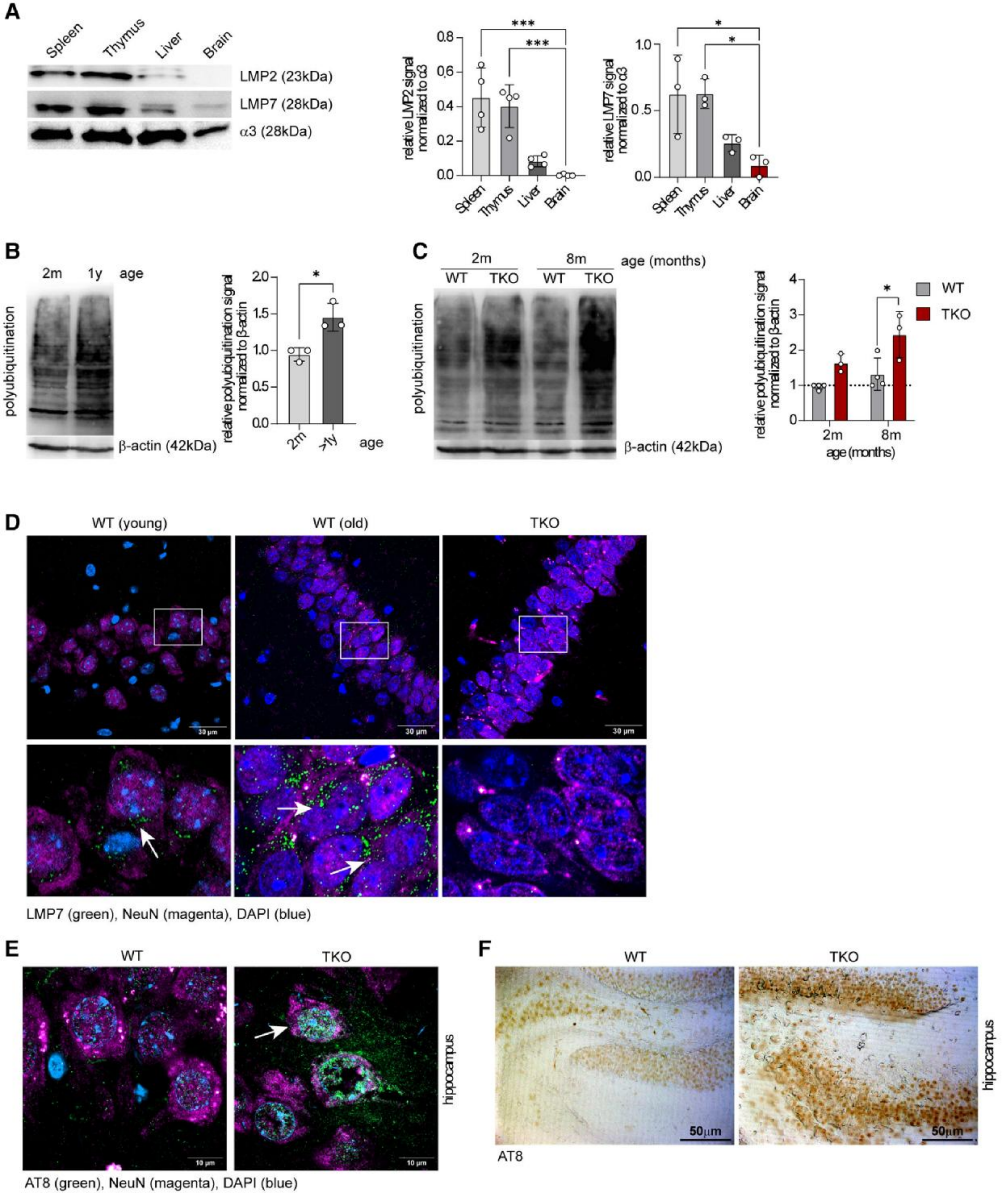
**Immunoproteasome expression in the brains of young and old mice.** (**A**) A western blot analysis of immunoproteasome distribution in the spleen, thymus, liver and brain of 2-month-old female WT mice. A representative western blot is shown (left side). The bar graphs display a relative abundance of low*-*molecular-weight protein 2 (LMP2) and low-molecular-weight protein 7 (LMP7) normalized to the proteasome subunit α3 (*n* = 3–4). The statistical analysis was performed by using one-way ANOVA. Data are presented as mean ± SD, *P = 0.01–0.05, ***P < 0.001. Uncropped blots are shown in Supplementary Fig. 6. (**B**) A western blot of polyubiquitination in the hippocampi of 2-month-old and 1-year-old female WT mice. A representative western blot is shown (left side), and the bar graphs display a relative polyubiquitin signal normalized to β-actin (*n* = 3). The statistical analysis was performed by using an unpaired *t*-test. Data are presented as mean ± SD, *P = 0.01–0.05. (**C**) A western blot of 2- and 8-month-old female WT and TKO mice displays an increase in the polyubiquitination of TKO hippocampi compared with WT hippocampi. A representative western blot is shown. The bar graphs display polyubiquitination levels normalized to β-actin (*n* = 3–4). Statistical significance for (**B**) and (**C**) was analysed by using an unpaired *t*-test. Data are presented as mean ± SD, *P = 0.01–0.05. Uncropped blots are shown in Supplementary Fig. 6. (**D)** A fluorescence staining of LMP7 (green) and NeuN (magenta, a neuronal marker) in the brains of young and old WT mice. A staining of the brains of old TKO mice is shown as a negative control. (**E**) A fluorescence staining of phospho-tau (AT8 = green, white arrows) in the CA3 region of the brains of WT versus TKO mice. The neuronal nuclear protein (NeuN, magenta) serves as a neuronal marker, and DAPI (blue) stains the nuclei. (**F**) A diaminobenzidine staining of phospho-tau (AT8) in the hippocampi of old WT and TKO mice (CA1).

The emerging role of immunoproteasomes in various non-immune cells prompted us to investigate the tissue-specific distribution of this enzyme in naïve WT mice. Previously, we examined the amount of immunoproteasomes in various organs and detected a high expression of immunoproteasomes in primary and secondary lymphoid organs, such as the thymus, spleen and small intestine.^15^ Interestingly, when we compared the mRNA and protein expressions of LMP2 and LMP7 in the spleen and thymus with those in the liver and brain in 2-month-old mice, we confirmed high levels of immunoproteasomes in tissue containing a high percentage of immune cells, while the immunoproteasome expression in the liver was low. The lowest amount of immunoproteasomes was found in the brain. This suggests that probably only a few immune cells are capable of expressing this enzyme in this largely immune-privileged organ (Fig. 1A and Supplementary Fig. 1A). When we compared young WT mice (2 months’ old) with aged WT animals (over 1 year old), we detected a strong accumulation of polyubiquitinated proteins in the brains of old mice, indicating the existence of age-related cellular stress (Fig. 1B). We also observed that aged animals had a tendency to induce immunoproteasomes in comparison with young mice (Supplementary Fig. 1B). Of note, by comparing young WT and immunoproteasome-deficient mice (TKO mice lacking all three immunoproteasome subunits), we found a strong increase in polyubiquitin conjugates in the brains of TKO mice when compared with WT animals (Fig. 1C). When we analysed the cell type–specific expression of LMP2 and LMP7 in old WT mice, we found a predominant abundancy of immunoproteasomes in two different cell types, neurons and microglia (Fig. 1D and Supplementary Fig. 1C). Of note, in young WT animals, the neuronal expression of immunoproteasomes was marginal (Fig. 1D).

Interestingly, we were not able to detect this altered accumulation of polyubiquitinated proteins in the brains of young LMP7-deficient mice, suggesting that the formation of mixed proteasomes containing LMP2 and MECL-1 can compensate for the lack of LMP7 (Supplementary Fig. 2A and B). To provide further evidence that TKO mice are prone to accumulate intracellular polyubiquitinated proteins upon cellular stress, we treated WT dendritic cells (DCs) expressing high amounts of immunoproteasomes and immunoproteasome-deficient DCs with hydrogen peroxide. We detected a delayed degradation of polyubiquitinated aggregates and a strong induction of C/EBP homologous protein, an apoptosis-mediating transcription factor and the essential component of the endoplasmatic reticulum (ER) stress response, in cells lacking immunoproteasomes (Supplementary Fig. 3A and B). Similarly, in the brains of young TKO mice, we observed increased ER stress–induced eIF2-α phosphorylation when compared with young WT animals (Supplementary Fig. 3C). Tau hyperphosphorylation, previously shown to be a sign of accelerated ageing and neuronal damage,^16^ was highly increased in neurons in several brain areas of old TKO mice (Fig. 1E and F and Supplementary Fig. 4A and B), further suggesting increased neuronal stress in aged TKO mice.

### Deficiency in immunoproteasomes results in the development of epilepsy during ageing

**Figure 2:**
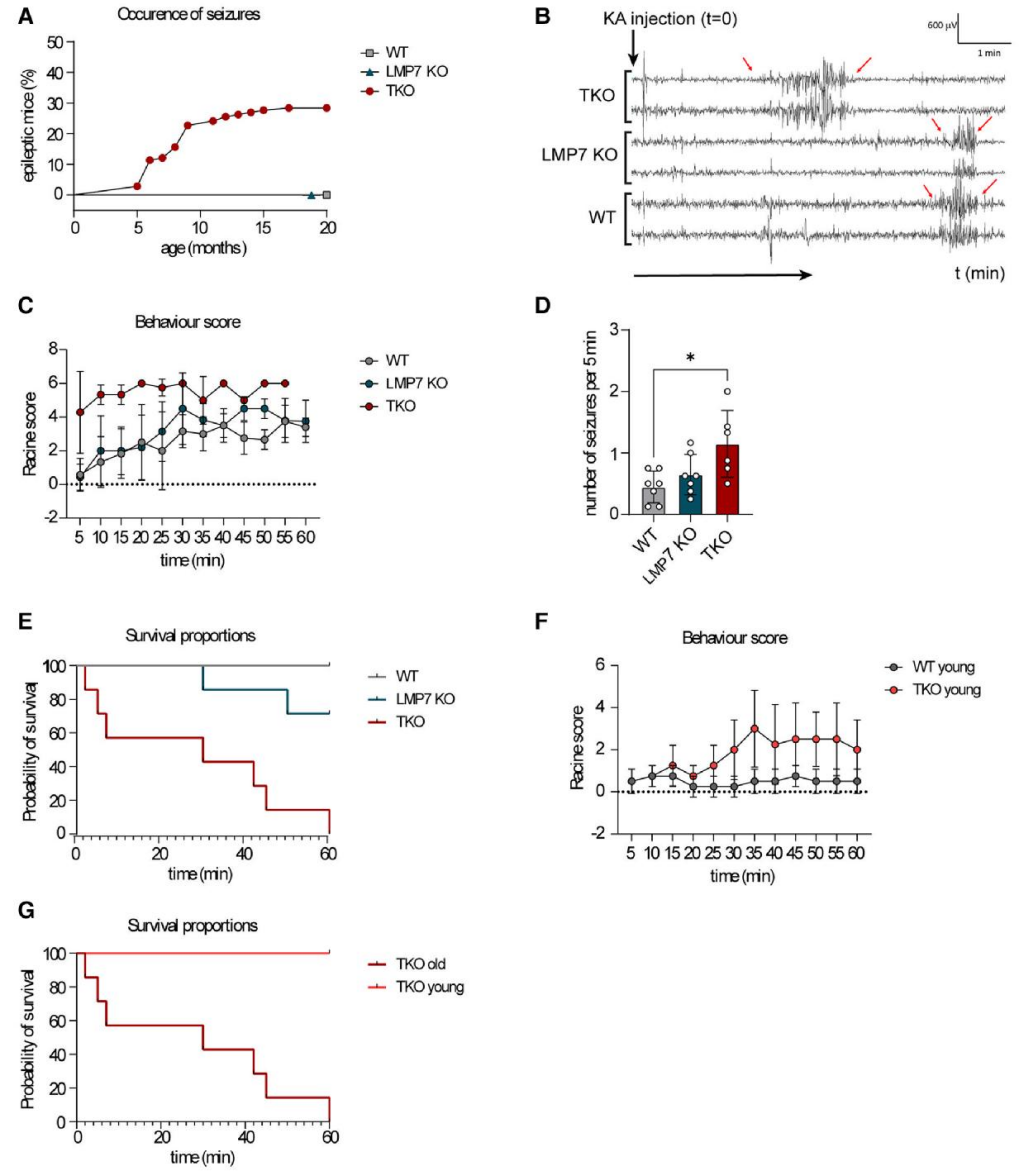
**Immunoproteasome deficiency increases excitability and the risk of developing epilepsy.** (**A**) TKO mice develop epileptic seizures at an age of 5–10 months. Female mice (*n* = 141) were scored over 20 months. The graph displays the occurrence of seizures in per cent. Statistical significance was analysed by performing a Mantel–Cox test ****P < 0.0001. (**B–G**) Seizure susceptibility of female TKO mice was tested via an i.p. injection of KA (10 mg/kg). The mice were monitored for 60 min after injection. (**B**) A representative EEG of female WT, LMP7 KO and TKO mice. (**C**) The Racine score was estimated in intervals of 5 min. Statistical significance was analysed by using one-way ANOVA (Turkey’s multiple comparisons test, *n* = 7 mice/group): WT versus LMP7 KO n.s., WT versus TKO ****P < 0.0001, LMP7 KO versus TKO ****P < 0.0001. (**D**) The number of seizures per 5 min was calculated by counting the occurrence of seizures over 40 min (*n* = 7 mice/group). Statistical significance was analysed by using one-way ANOVA: WT versus LMP7 n.s., LMP7 versus TKO n.s., WT versus TKO *P = 0.0122. (**E**) The graph displays the survival of animals (*n* = 7 mice/group). Statistical significance was analysed by using the Mantel–Cox test: WT versus TKO ****P < 0.0001, WT versus LMP7 KO n.s., LMP7 KO versus TKO **P = 0.0031. (**F**) The behavioural score of young female WT and TKO animals after an injection of KA (*n* = 4 mice/group). Statistical significance was analysed by performing a paired *t*-test. ***P = 0.0001. (**G**) The Survival of young and old female TKO mice after an injection of KA (*n* = 4–7 mice per group). Statistical significance was analysed by using the Mantel–Cox test **P = 0.003.

Our results suggest that aged mice might need a functional shift of proteasome composition in the brain towards induced generation of immunoproteasome to be able to handle the cellular and metabolic stress. Consequently, the lack of immunoproteasomes may, in turn, lead to neurological deficits. Previously, we showed that increased polyubiquitination is a molecular feature of SE induced by an intra-amygdala injection of KA, which is an experimental model for the most common form of epilepsy in adults, TLE.^17^ This finding suggests a causal relationship between neuronal excitability and accumulation of polyubiquitinated conjugates in the hippocampus. Indeed, TKO mice completely lacking immunoproteasome, but not LMP7-deficient animals, developed recurrent epileptic seizures, starting from the age of 6 months (Fig. 2A and Supplementary Video 1). These data indicate an existence of a molecular threshold for controlling the oxidative and proteotoxic stress in the aged brain. It appears that the lack of one, but not that of three immunoproteasome subunits, is still able to cope with accumulated, damaged proteins.

**Figure 3:**
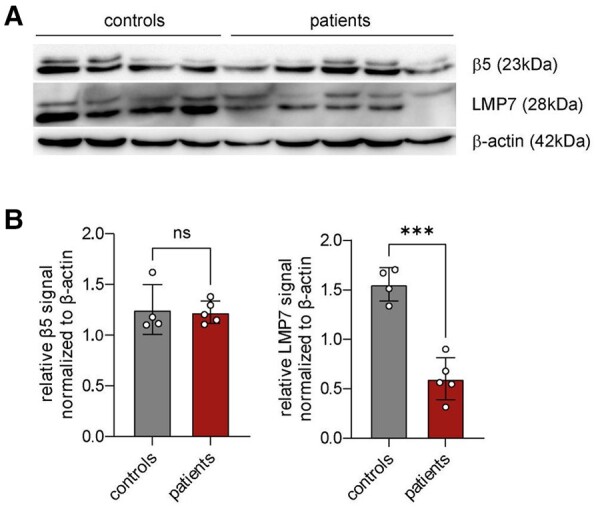
**An analysis of immunoproteasome expression in patients with TLE.** (**A**) A western blot analysis of immunoproteasome subunit LMP7 and constitutive proteasome subunit β5 in female patients with epilepsy (*n* = 5) compared with controls (*n* = 4). (**B**) The bar graphs show relative β5 and LMP7 signals normalized to β-actin. The statistical analysis was performed by using an unpaired *t*-test (n.s. = not significant; ***P < 0.001). Data are presented as mean ± SD. Uncropped blots are shown in Supplementary Fig. 6.

To further confirm a lowered seizure threshold in TKO mice, 1-year-old WT, LMP7 KO and TKO animals were injected with a low dose of KA (10 mg/kg) intraperitoneally (i.p.). This caused first seizure bursts in WT and LMP7 KO mice ∼10–20 min post-KA injections (Fig. 2B). In contrast, and confirming an increased susceptibility to KA, TKO mice experienced their first seizure burst already within the first 5 min post-KA injections (Fig. 2B). Moreover, TKO mice showed more severe epileptic seizures during a 60 min recording period post-KA as measured via behaviour changes and EEG (Fig. 2C and D), and an increased mortality with all TKO mice dying within 60 min after i.p. KA injection (Fig. 2E). Of note, LMP7-deficient mice displayed an intermediate phenotype (Fig. 2E), indicating a defective proteolysis in the brains of these animals, which, however, was not capable of provoking spontaneous seizures at the steady state (Fig. 2A). Further confirming a lowered seizure threshold in TKO mice, 2-month-old TKO mice subjected to i.p. KA experienced more severe seizures when compared with age-matched WT mice (Fig. 2F). Notably, while aged TKO mice reached a fatal SE within 1 h, all young TKO animals survived the same i.p. dose of KA (10 mg/kg; Fig. 2G). This finding supports our hypothesis that the proteolysis of age-related polyubiquitinated protein aggregates is disturbed in the absence of immunoproteasomes, which, in turn, drives the increase in local neuronal excitability. To test whether the expression of immunoproteasomes was altered during human epilepsy, we analysed the abundancy of this enzyme in the resected brain tissue of patients with TLE. In contrast to the normal abundance of the constitutive proteasomal subunit β5, the immunoproteasome subunit LMP7 was present at lower levels in all cortical samples of patients with TLE when compared with the autopsy control cortex (Fig. 3A and B).

### Increased anxiety and ataxia in aged immunoproteasome-deficient mice

**Figure 4:**
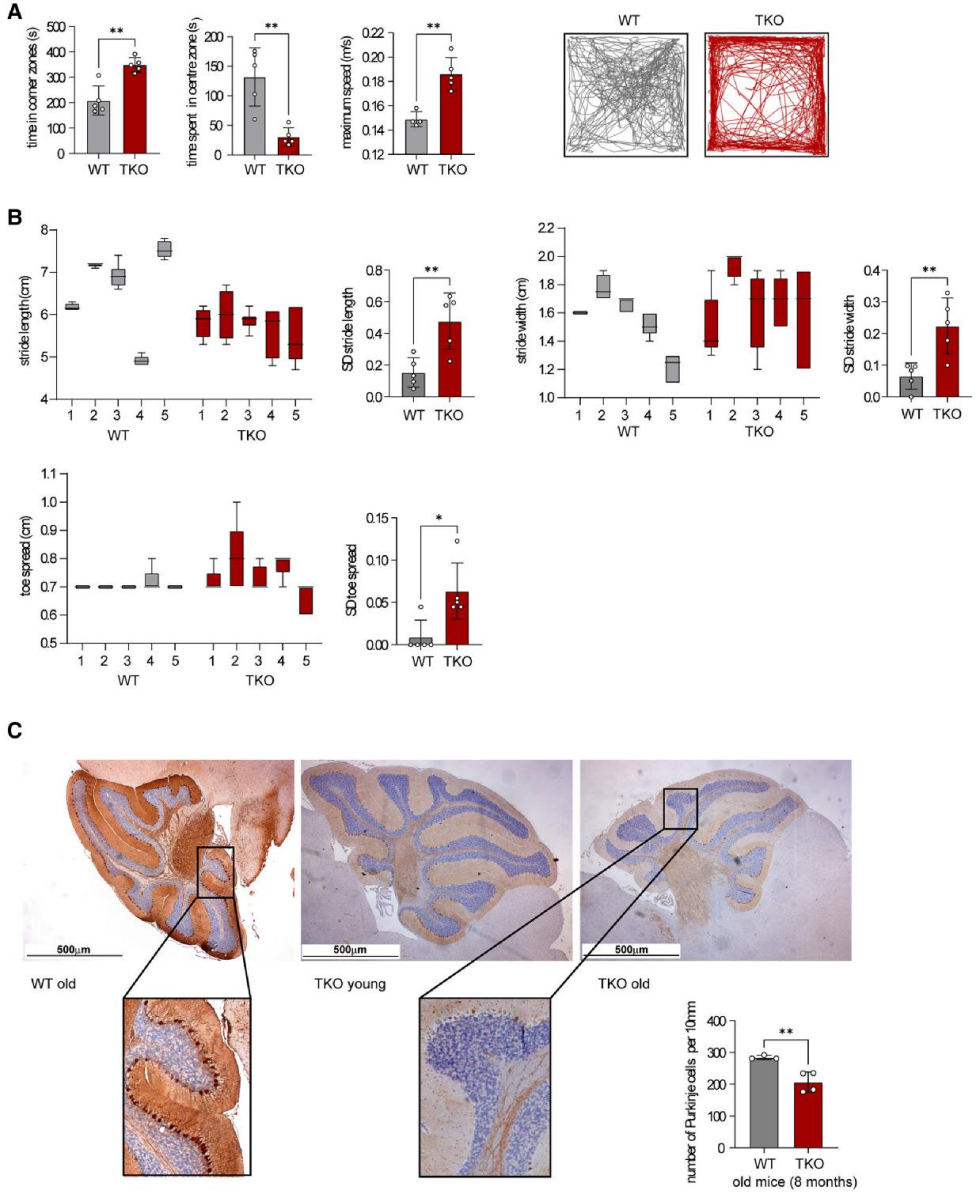
**TKO animals display several neurological disorders apart from recurrent seizures.** (**A**) Female TKO animals show increased anxiety in the open field study compared with age-matched WT mice (>1-year-old mice; *n* = 5 mice/group). The statistical analysis was performed by using an unpaired *t*-test. (**B**) A gait analysis of >1-year-old female TKO and WT mice (*n* = 5 mice/group). The stride length, stride width and toe spread were measured. The SD value of each mouse was calculated. (**C**) Calbidin staining in female WT and TKO mice shows a significant loss of Purkinje cells in TKO animals (*n* = 4 mice/group). The number of Purkinje cells per 10 mm was calculated. The statistical analysis was performed by using the unpaired *t*-test. *P = 0.01–0.05; **P = 0.001–0.01.

In addition to alterations in brain hyperexcitability, increased polyubiquitination seen in aged TKO mice most likely impacts several crucial cellular processes. Therefore, to test whether aged TKO mice show other behaviour changes in addition to epileptic seizures, 1-year-old mice were subjected to the open field test, one of the most commonly used test to measure behaviours in animal models. It was found that the TKO mice had more entries into the corner and spent more time in the corner zones when compared with WT mice (Fig. 4A), suggesting an anxiety-like behaviour. Of note, the TKO mice also showed a higher speed during the 10 min they spent in the open field (Fig. 4A), suggesting hyperactivity. We found a similar, although not as pronounced, trend for LMP7 KO mice (Supplementary Fig. 5A), in line with a previous study reporting behaviour deficits in such animals.^18^ Next, to investigate whether the TKO mice showed any alterations in locomotor activity, 1-year-old TKO mice and age-matched WT mice were subjected to a gait analysis. The generated metrics show a higher variance in the stride length, width and toe spread of TKO animals compared with those of WT mice (Fig. 4B and Supplementary Fig. 5B), suggesting gait problems in TKO mice. The cerebellum is the main brain region controlling movement. Of note, a histological analysis of the cerebellum of TKO mice revealed increased tau phosphorylation (Supplementary Fig. 4A and B) and a pronounced lack of Purkinje cells (Fig. 4C), which may explain the observed locomotor deficits in immunoproteasome-deficient mice (Supplementary Video 2). Together, our results suggest that a functional immunoproteasome is critical to maintain a healthy brain during ageing.

## Discussion

The activity of immunoproteasomes is required to efficiently eliminate misfolded and defective proteins,^9^ which is a cellular prerequisite for the maintenance of protein homeostasis. Previously, we demonstrated that this enzymatic complex is involved in the immunopathogenesis of a chronically inflamed intestine.^19,20^ Paradoxically, while this protease seems to act as a central mediator of the inflammation-driven neoplasia,^21,22^ here, we suggest a critical role for immunoproteasomes during healthy brain ageing.

Due to the increasing lifespan, age is becoming an important risk factor for a plethora of pathological conditions, particularly for diseases of the brain. This includes neurodegenerative diseases such as Alzheimer’s disease and epilepsy, particularly prevalent among the elderly.^23,24^ Possible reasons among others include mitophagy, cellular senescence, genomic instability and protein aggregation. With the ageing population, age-related diseases are putting the healthcare system under immense pressure, and therefore, much effort is invested in identifying possible risk factors contributing to age-related diseases.

Our results suggest age-related changes in immunoproteasome function as an additional risk factor. Here, we show age-dependent alterations in brain immunoproteasome expression in line with previous studies.^25,26^ We also show that the deficiency in immunoproteasomes was accompanied by an increase in the amount of polyubiquitinated proteins and the emergence of epilepsy and other behaviour alterations (i.e. anxiety and ataxia). According to the cooperative model for immunoproteasome assembly, the LMP7 subunit has a central role, as the lack of this subunit impairs the incorporation of LMP2 and MECL-1 into a newly synthetized proteasome complex.^27^ Recently, mice deficient in LMP7 were shown to have an impairment in proteotoxic stress related to defective microglial function.^28^ However, only a complete deficiency of all three immunoproteasome subunits results in age-related epileptic seizures, suggesting that mice lacking LMP7 still have a relatively functional ubiquitin–proteasome system.

Recent studies have demonstrated that the neurologic innate immunity of the CNS may be implicated in the development of seizures,^29,30^ suggesting a link between inflammation and epilepsy. Interestingly, previous reports suggested that the immunoproteasome was one of the contributors to the disease phenotype and that its inhibition was protective. This includes inhibiting the LMP7 subunit in epilepsy.^11^ In this study, we observe that mice deficient in all three immunoproteasome subunits show increased brain hyperexcitability. This suggests that, whereas LMP7 may contribute to pathological changes in the brain such as seizures, the remaining subunits are necessary for normal brain functioning. Another possibility is that, while a short inhibition of the immunoproteasome may provide beneficial effects, a prolonged suppression of immunoproteasome function may lead to deleterious effects for normal brain functioning. This is further evidenced by the selective loss of Purkinje cells, in line with a recent study suggesting that reduced proteasome activity in ageing brains is a driver of neurodegeneration.^31^

The cellular mechanisms of how the immunoproteasome protects the brain remain to be established. In this study, we show increased tau phosphorylation in aged TKO mice throughout the brain. That the hyperphosphorylation of tau contributes to neurodegeneration is well established.^32^ Mounting evidence suggests tau phosphorylation to contribute to seizures and epilepsy.^33^ Of note, tau hyperphosphorylation is also a known sign of brain ageing.^16^ Whether tau phosphorylation is a causative factor of how the lack of the immunoproteasome contributes to neurological deficits or is a consequence of immunoproteasome-induced pathology, however, remains to be determined via, for example, the use of tau-deficient mice. The immunoproteasome is an important regulator of MHC-I molecules.^34^ MHC-I protein levels, while being highest in the brain during neonatal development, decrease during adulthood, but increase again during ageing.^35^ The MHC-I complex has been shown to be involved in neuronal differentiation, synapse formation and synapse density,^36^ possibly contributing to increased hyperexcitability seen in our TKO mice. In addition, mice lacking LMP2 or LMP7 display substantial immunological deficiencies that may also contribute to the observed neurological phenotype. Recent studies suggest that both LMP7-deficient mice and LMP2-deficient rats have some neurobehavioural dysfunctions,^18,37^ which may be even more pronounced in the absence of all three immunoproteasome subunits. Taken together, we conclude that all three immunoproteasome subunits, LMP2, MECL-1 and LMP7, are required in order to effectively protect the ageing brain from neurological disorders.

For our study, we used a genetic approach to decipher the function of the proteasome, which is unrealistic as a therapeutic scenario. However, the aim of this study was to investigate the fundamental role of the immunoproteasome in normal brain functioning and whether it impacts brain pathology during the process of ageing. Future studies should be designed to test whether pharmacological targeting of the immunoproteasome leads to unwanted side effects similar to what has been observed in our study, particularly longer treatment regimes, which would be required for chronic diseases such as Alzheimer’s disease or epilepsy.

## Supplementary material

Supplementary material is available at *Brain Communications* online.

## Supplementary Material

fcae017_Supplementary_Data

## Data Availability

All data and materials used for this research project will be made available upon reasonable request.
