# Supplementary material for: Immunoproteasome deficiency results in age-dependent development of epilepsy
Source: Brain Commun. 2024 Jan 29;6(1):fcae017. doi: 10.1093/braincomms/fcae017 (PMC10839634; doi:10.1093/braincomms/fcae017)
Supplement: fcae017_Supplementary_Data [file fcae017_supplementary_data.zip › Supplementary_material.docx]

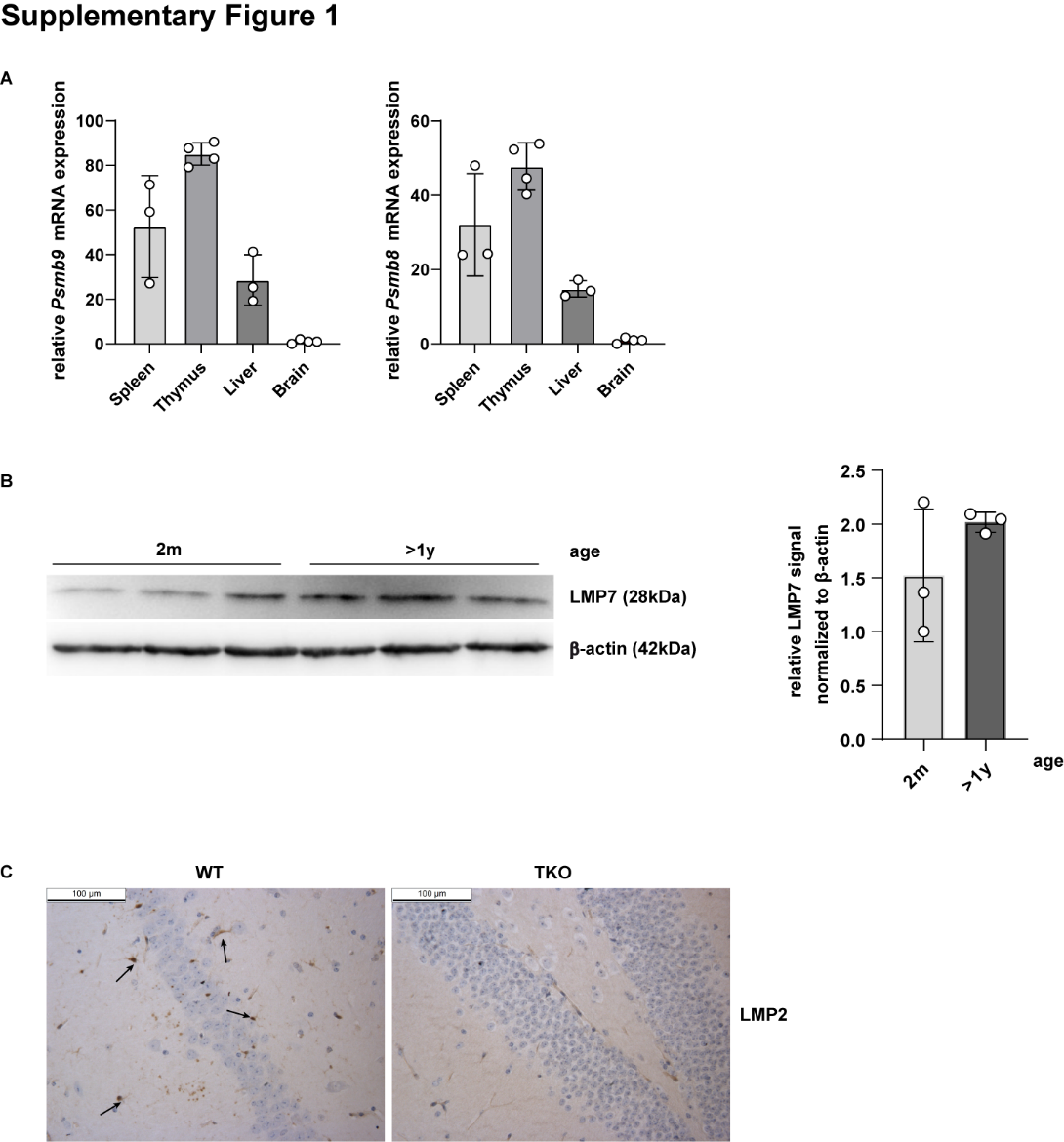


**Supplementary Figure 1 Analysis of immunoproteasome expression in WT brains.** (**A**) RT-qPCR analysis of immunoproteasome subunit Psmb8 (LMP7) and Psmb9 (LMP2) expression in spleen, thymus, liver and brain. Bar graphs represent n=3-4 independent experiments. (**B**) Western blot analysis of 2-month- and > 1-year-old WT hippocampi for LMP7 subunit. Bar graph represents relative LMP7 signal normalized to β-actin (n=3). Statistical significance was tested via unpaired t-test. (**C**) Immunohistological staining of LMP2 subunit in WT brain (1 year old mice). TKO mice serves as negative control.


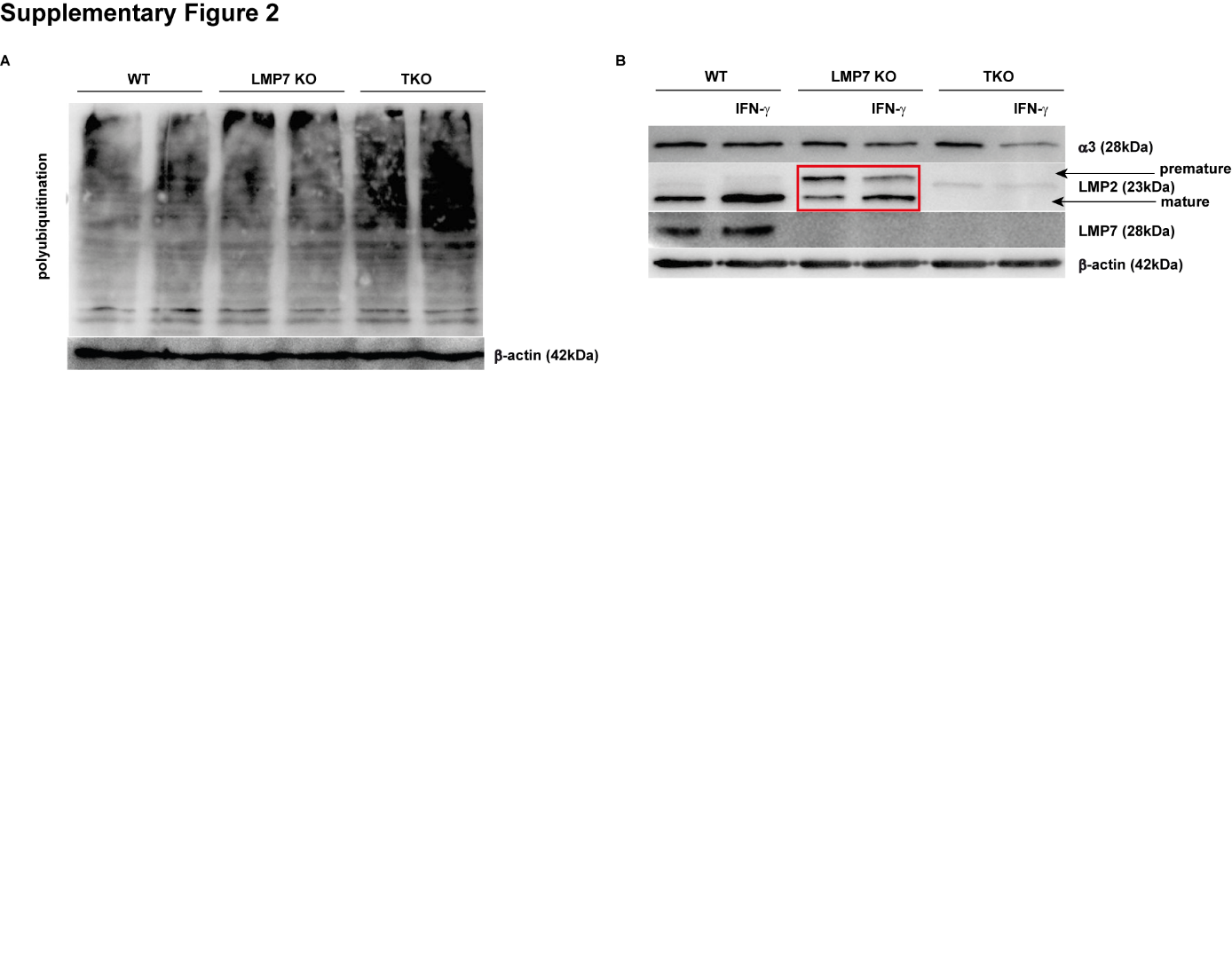


**Supplementary Figure 2 Mixed proteasomes prevent enhanced polyubiquitination in aged LMP7 KO hippocampi.** (**A**) Western blot analysis of WT, LMP7 KO and TKO hippocampi for polyubiquitination (2 months old mice). β-actin was used as loading control. One of three independent experiment is shown. (**B**) Western blot analysis of α3, LMP2 and LMP7 in WT, LMP7 KO and TKO BMDCs. β-actin was used as loading control.


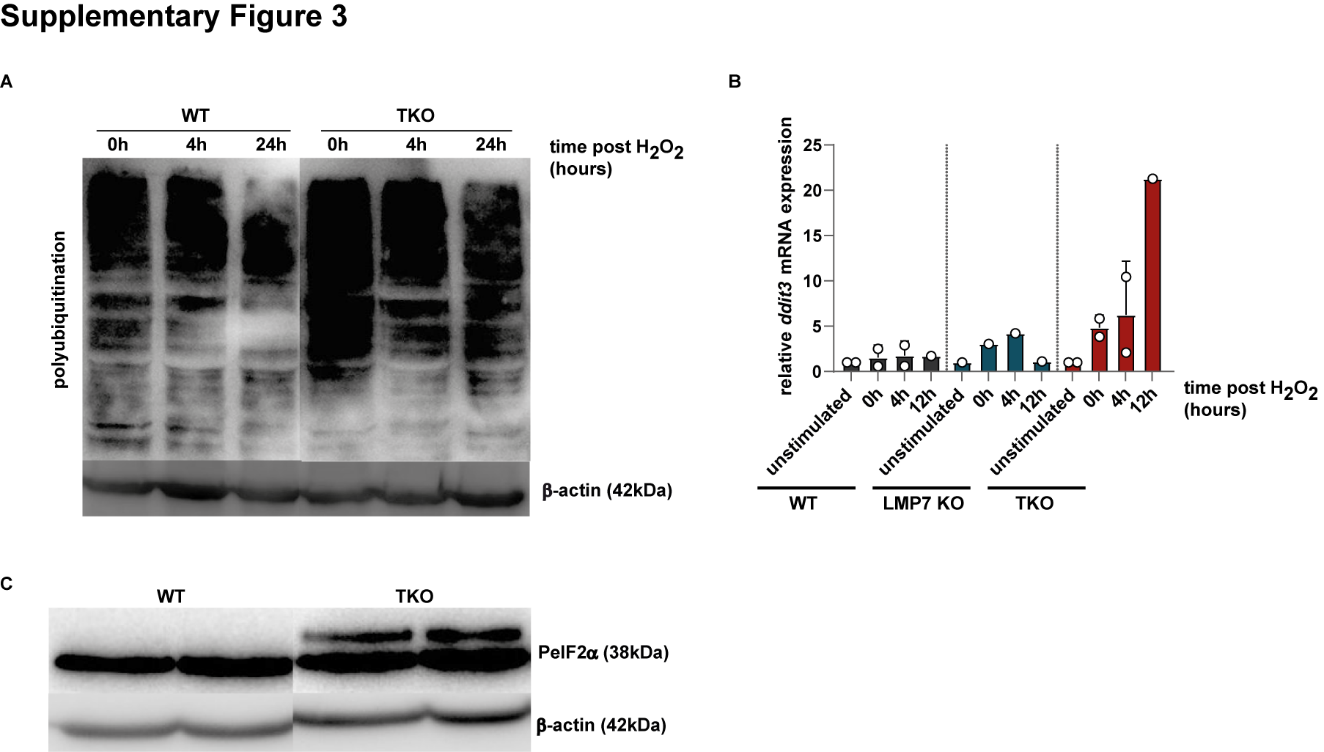


**Supplementary Figure 3** **Immunoproteasome-deficient cells induce unfolded protein response (UPR) signalling**. (**A** and **B**) BMDCs derived from WT and TKO mice were pre-incubated with IFN-γ for 48h. Afterwards, the cells were treated with H_2_O_2_ to induce ER stress. After a washing step, cells were harvested at different time points and analysed via western blot and RT-qPCR. (**A**) Western blot analysis of DCs shows polyubiquitination at different time points after H_2_O_2_ treatment. (**B**) Bar graph shows the relative mRNA expression of ddit3 (CHOP) in DCs analysed by RT-qPCR after H_2_O_2_ treatment at different time points. (**C**) Western blot analysis of 2-month-old WT and TKO hippocampi for eIF2α phosphorylation. β-actin was used as loading control.


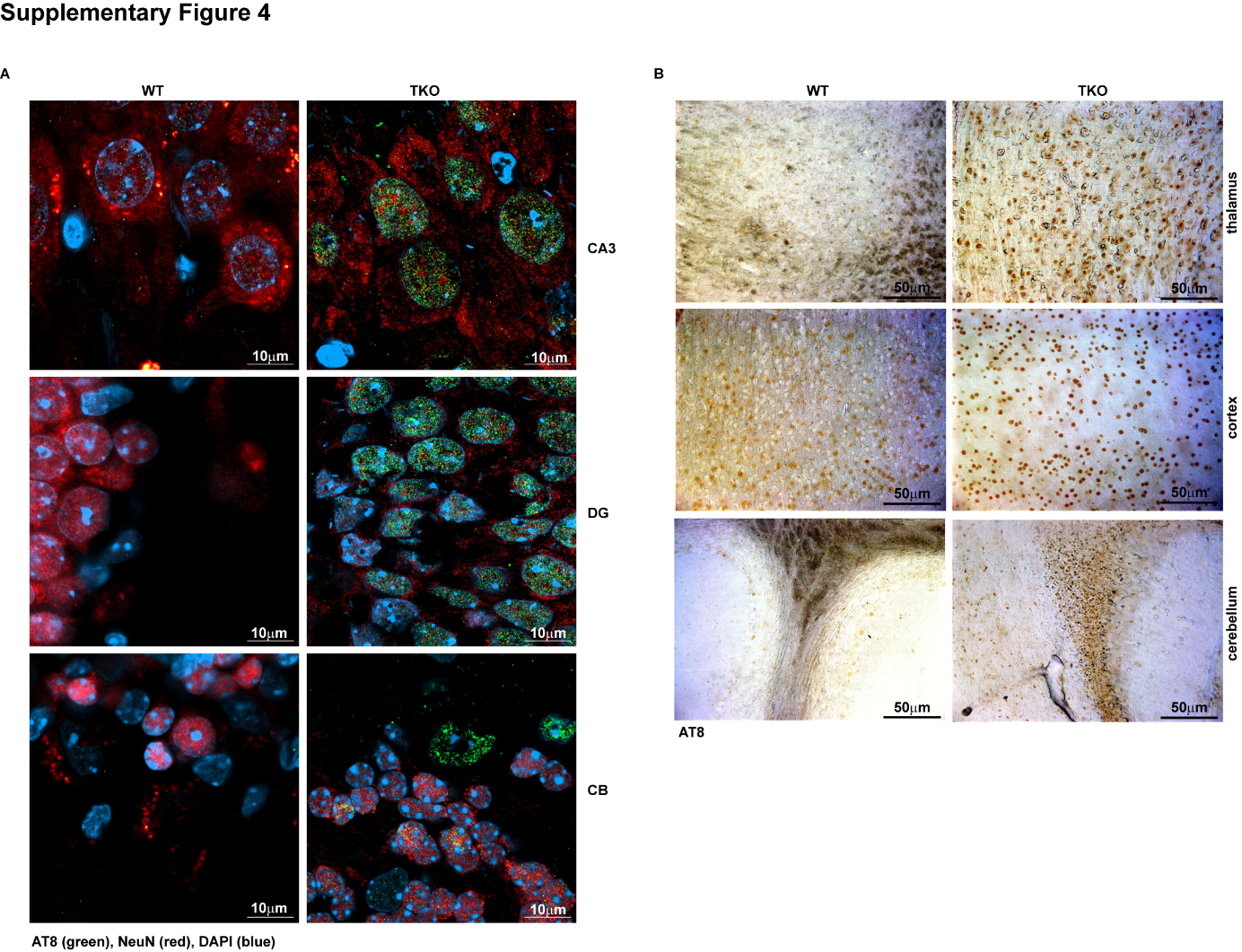


**Supplementary Figure 4 Phospho-tau accumulation in aged TKO brain regions. (A)** Fluorescence staining of different brain regions (CA3, DG and CB (cerebellum)) of WT and TKO mice for phospho-tau (AT8 = green), a neuronal marker (NeuN = red) and DAPI (blue). Representative images are shown (n=3 brains/group, 1 year old mice). (**B**) DAB staining of phospho-tau (AT8) in thalamus, cortex and cerebellum of aged WT and TKO mice (>1y).


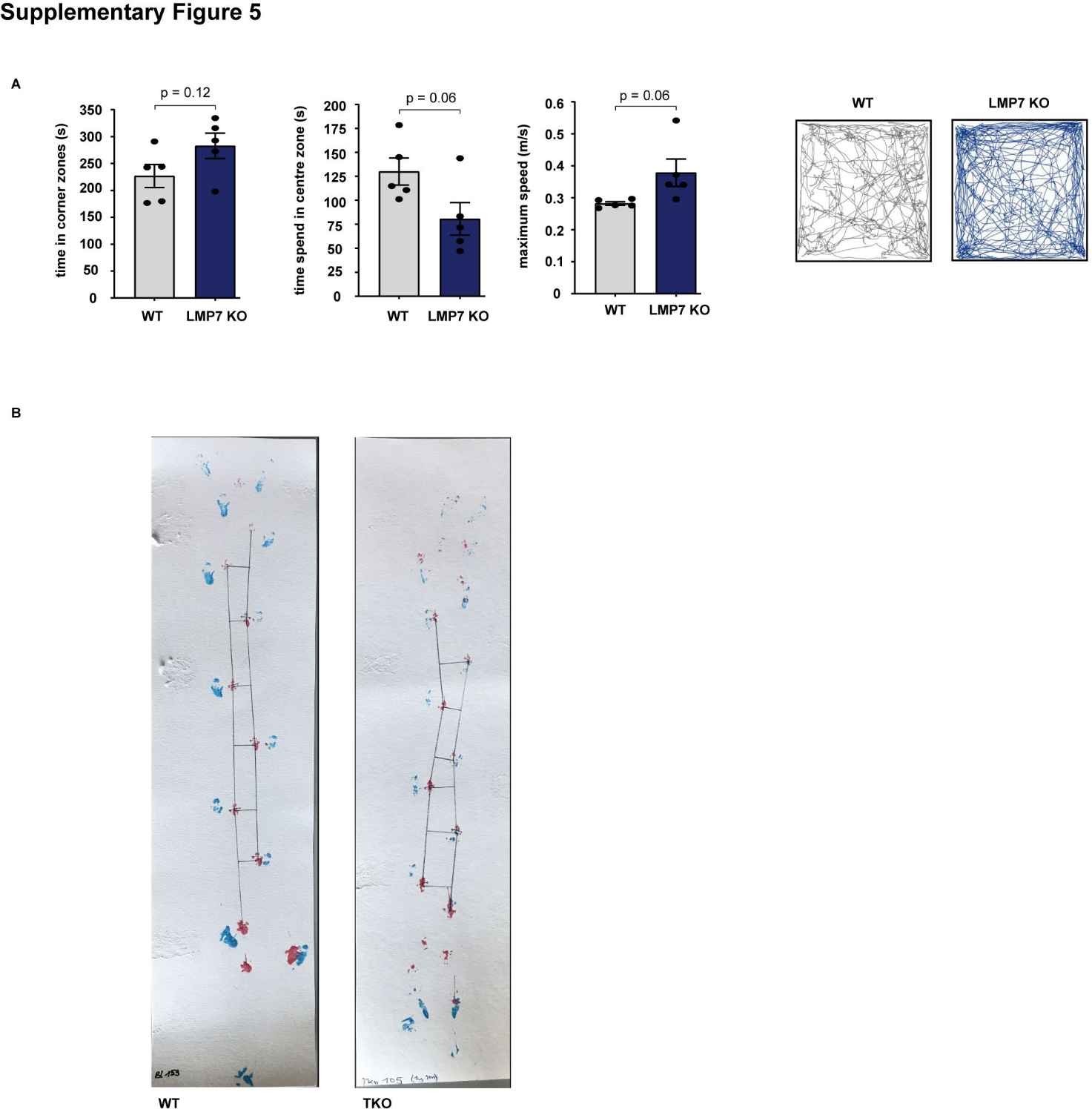


**Supplementary Figure 5 Open field study and** **gait analysis of old WT and immunoproteasome-deficient animals**. (**A)** Open field analysis of old female LMP7 KO and WT mice (n=5 mice per group). (**B)** A representative picture of gait analysis for female TKO mice and WT animals (n=5 mice per group) is shown. Forelimbs are coloured in red and hindlimbs in blue. The stride length, stride width and toe spread are marked.

**Supplementary Figure 6: Uncropped blots/gels for Figures 1 and 3 and Supplementary Figures 1, 2 and 3.**


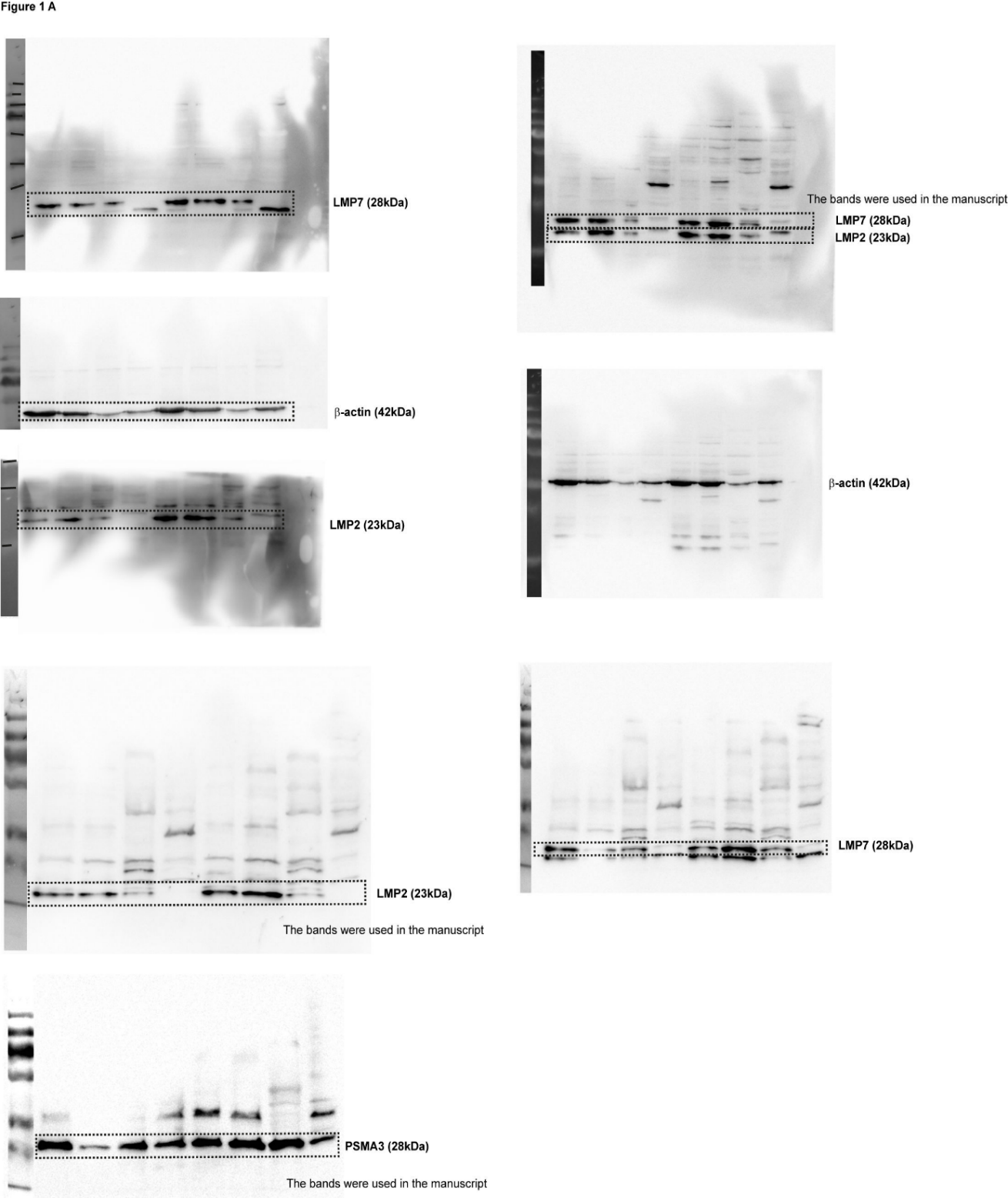


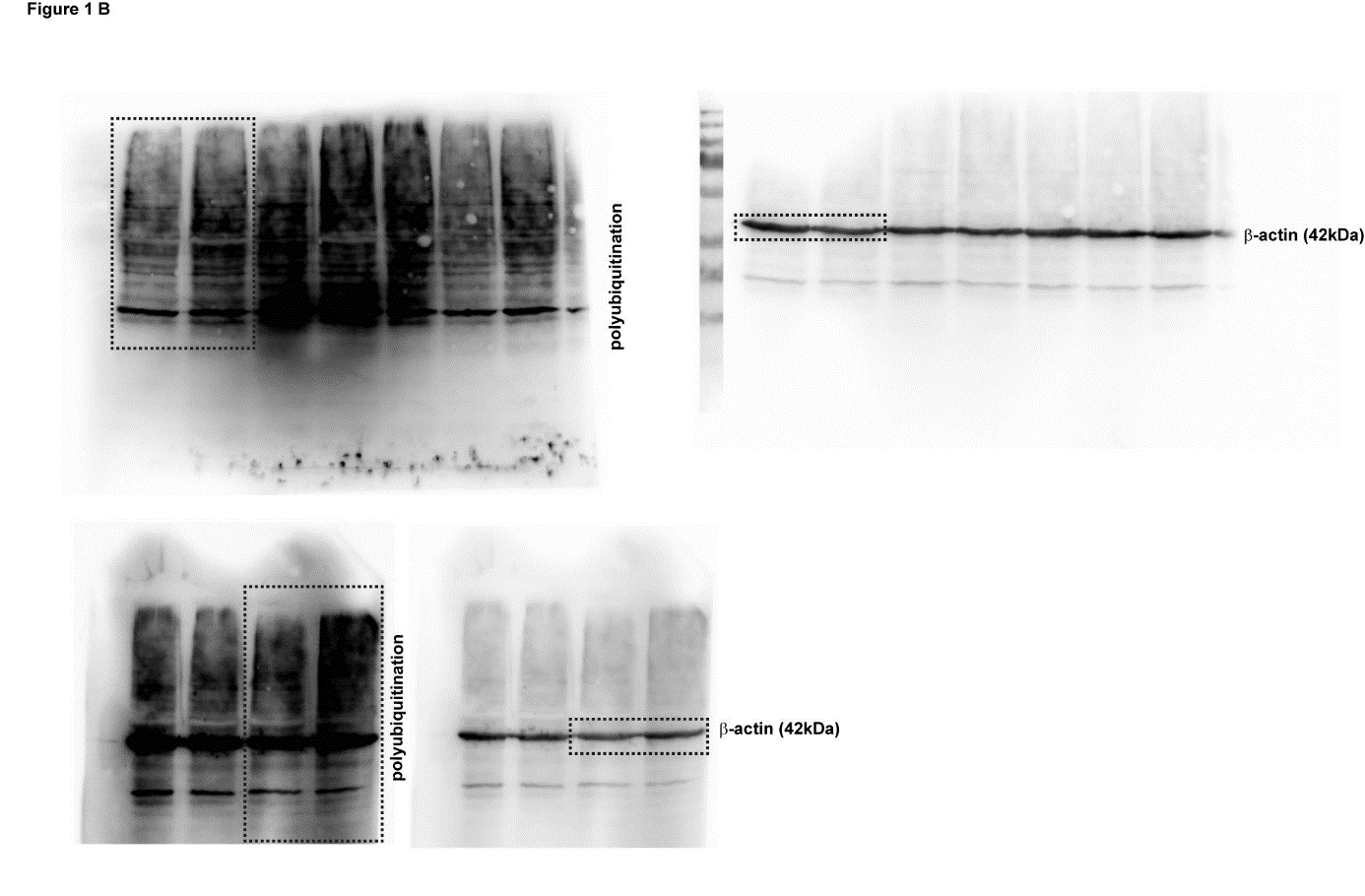

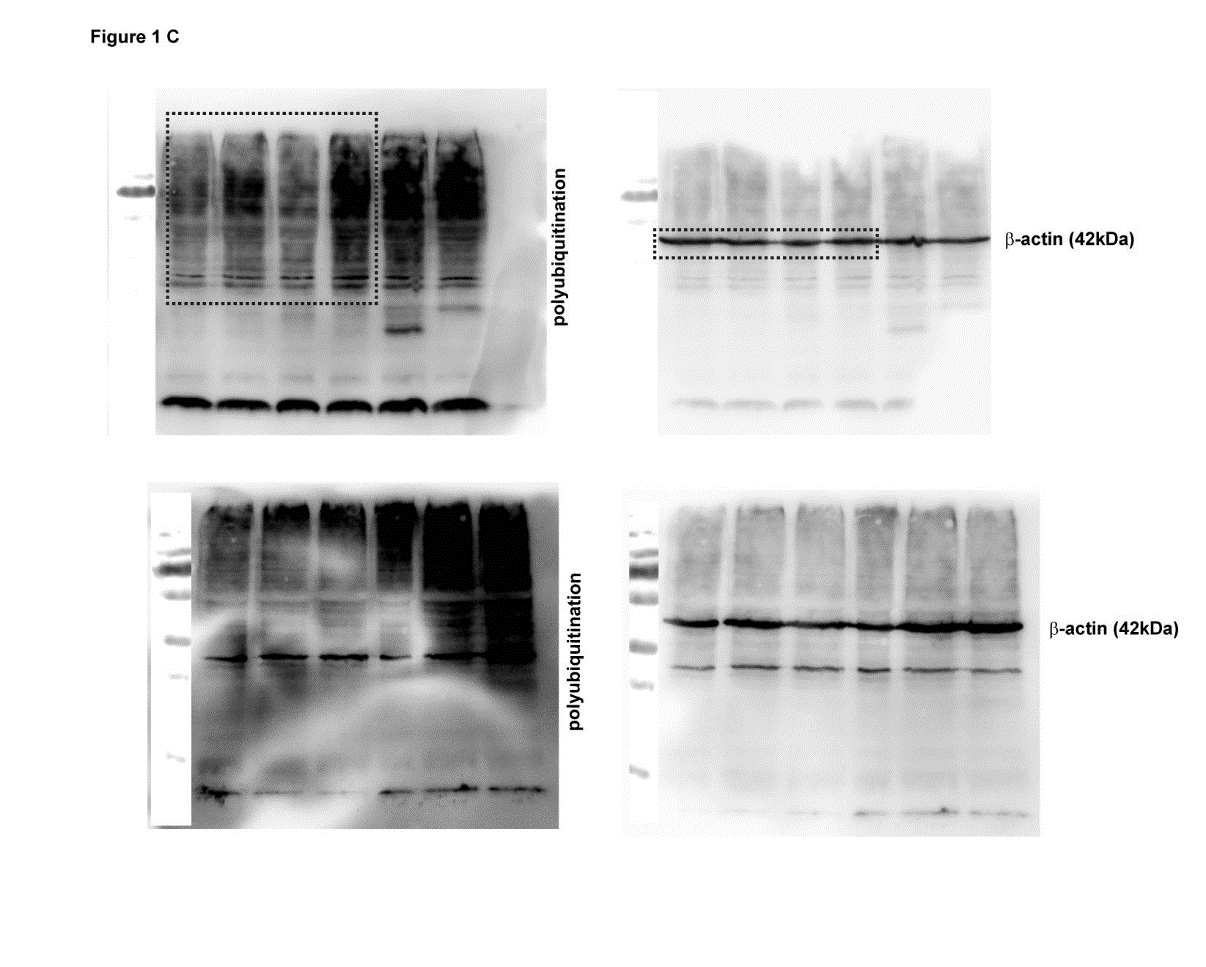


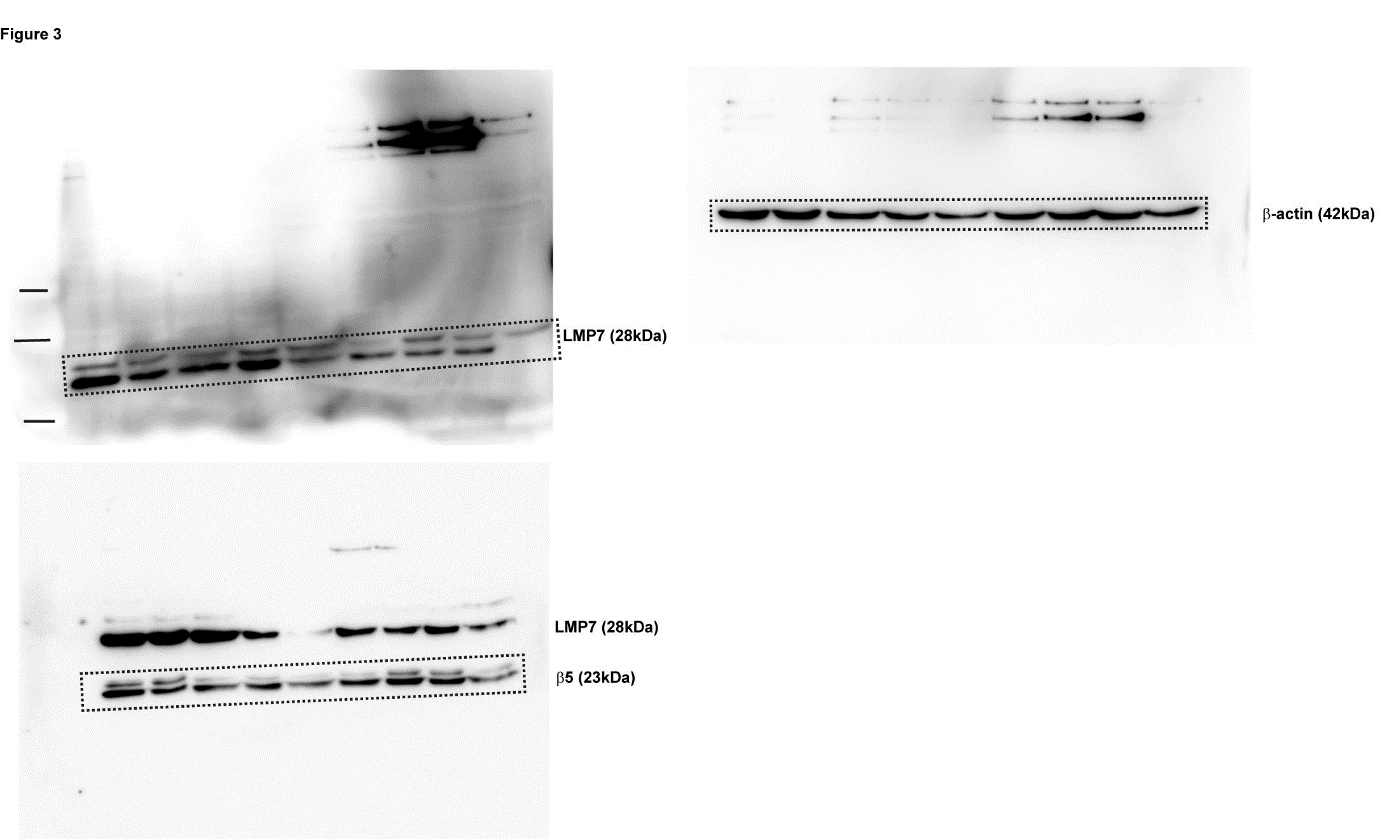


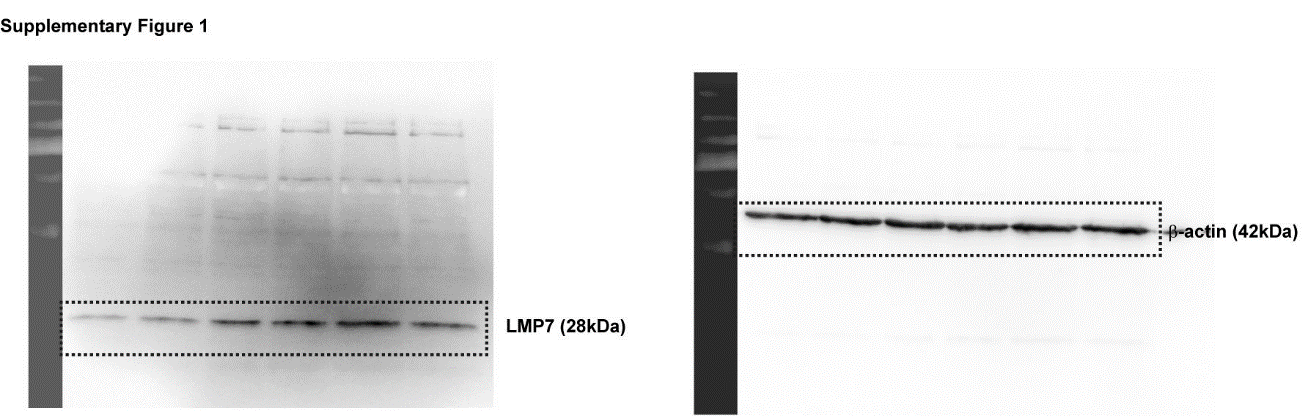

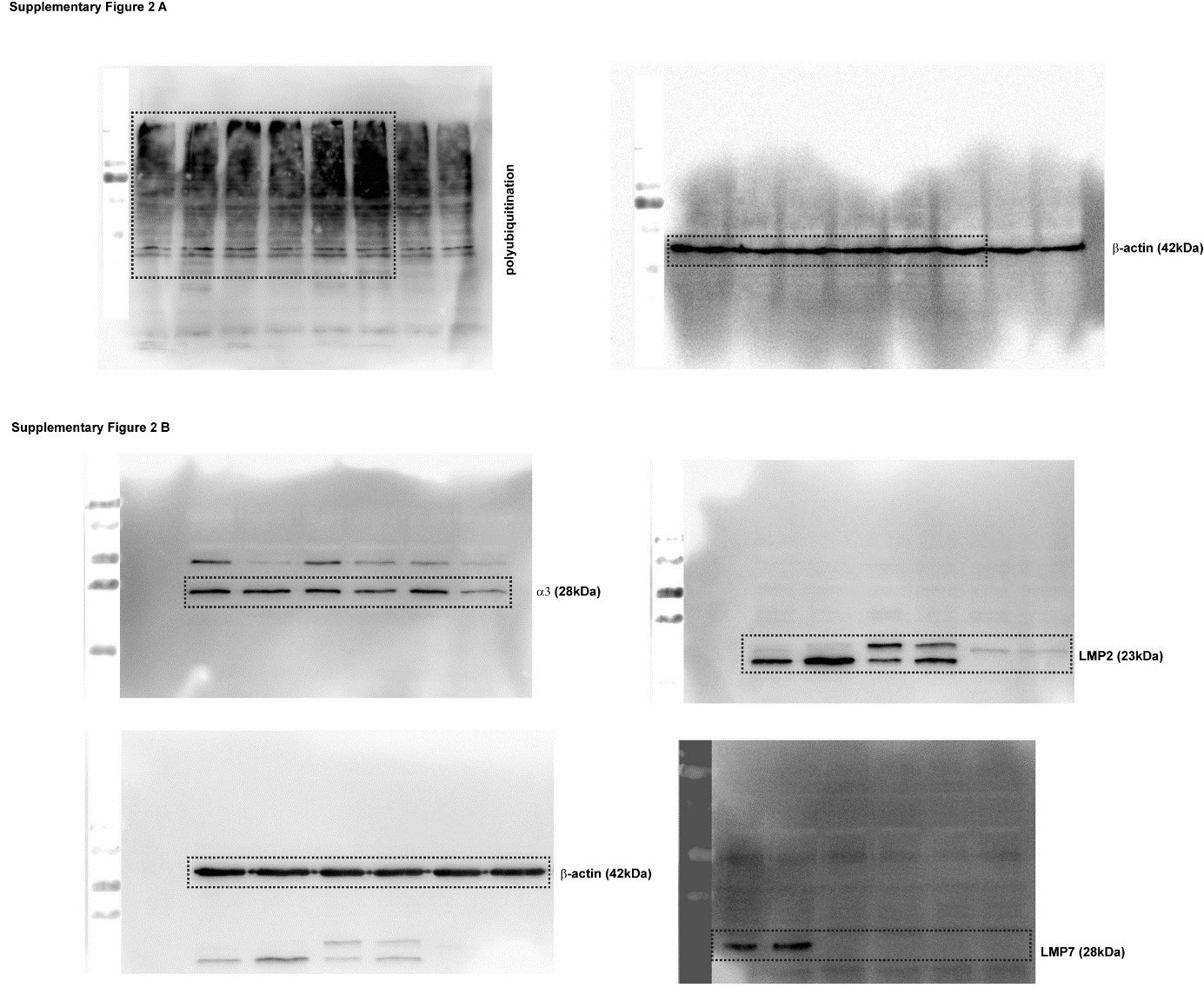


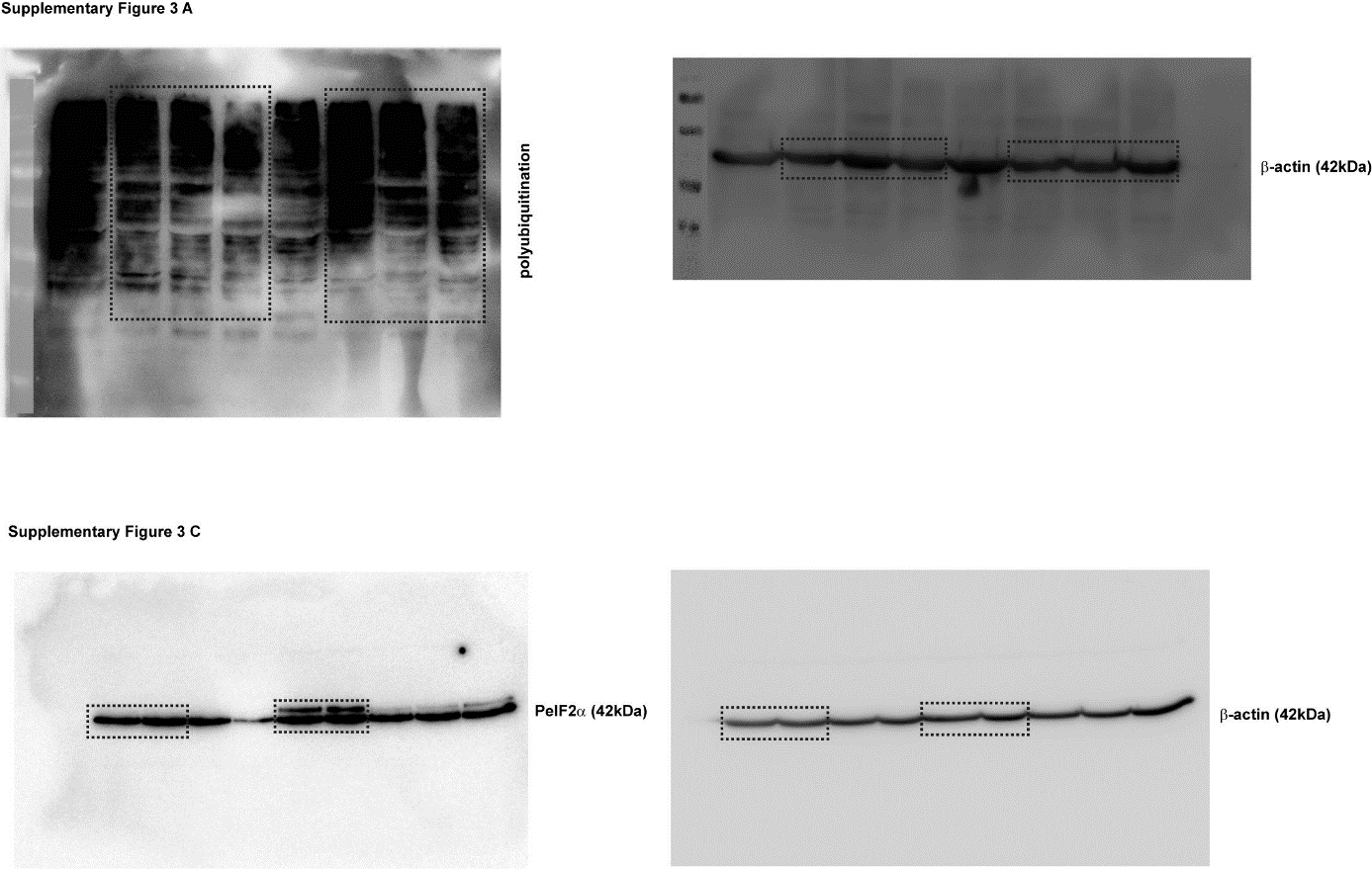


**Supplementary Table 1: Human brain samples**

| **ID code** | **Tissue** | **Age** | **Sex** | **Status** |
| --- | --- | --- | --- | --- |
| 5446 | BA21, temporal cortex | 17 | Female | control |
| 5538 | BA21, temporal cortex | 19 | Female | control |
| 1937 | BA21, temporal cortex | 23 | Female | control |
| 5579 | BA21, temporal cortex | 25 | Female | control |
| 5644 | BA21, temporal cortex | 29 | Female | control |
| 6302 | BA21, temporal cortex | 34 | Female | control |
| 1156 | BA21, temporal cortex | 45 | Female | control |
| 5611 | BA21, temporal cortex | 50 | Female | control |
| 5451 | BA21, temporal cortex | 57 | Female | control |
| 5997 | BA21, temporal cortex | 20 | Male | control |
| 6096 | BA21, temporal cortex | 28 | Male | control |
| 4287 | BA21, temporal cortex | 31 | Male | control |
| 6056 | BA21, temporal cortex | 37 | Male | control |
| 4645 | BA21, temporal cortex | 39 | Male | control |
| 5986 | BA21, temporal cortex | 41 | Male | control |
| 6058 | BA21, temporal cortex | 43 | Male | control |
| 5917 | BA21, temporal cortex | 49 | Male | control |
| 1578 | BA21, temporal cortex | 53 | Male | control |
| 5393 | BA21, temporal cortex | 56 | Male | control |
| 134 | neocortex | 55 | Male | patient |
| 135 | neocortex | 44 | Male | patient |
| 144 | neocortex | 22 | Male | patient |
| 160 | neocortex | 41 | Female | patient |
| 162 | neocortex | 52 | Female | patient |
| 122 | neocortex | 34 | Female | patient |
| 123 | neocortex | 54 | Male | patient |
| 128 | neocortex | 37 | Male | patient |
| 129 | neocortex | 36 | Female | patient |
| 131 | neocortex | 54 | Female | patient |

**Supplementary Video 1: Epileptic seizures in TKO mice**

**Supplementary Video 2: Ataxia in TKO mice**
